# Surface L-type Ca^2+^ channel expression levels are increased in aged hippocampus

**DOI:** 10.1111/acel.12157

**Published:** 2013-10-01

**Authors:** Félix Luis Núñez-Santana, Myongsoo Matthew Oh, Marcia Diana Antion, Amy Lee, Johannes Wilhelm Hell, John Francis Disterhoft

**Affiliations:** 1 Department of Physiology, Feinberg School of Medicine, Northwestern UniversityChicago, IL, 60611, USA; 2 Departments of Molecular Physiology and Biophysics, Otolaryngology-Head and Neck Surgery, and Neurology, University of IowaIowa City, IA, 52242, USA; 3 Department of Pharmacology, University of CaliforniaDavis, CA, 95615, USA

**Keywords:** biotinylation, Ca_v_1.2, Ca_v_1.3, calcium, phosphorylation, qRT-PCR

## Abstract

Age-related increase in L-type Ca^2+^ channel (LTCC) expression in hippocampal pyramidal neurons has been hypothesized to underlie the increased Ca^2+^ influx and subsequent reduced intrinsic neuronal excitability of these neurons that lead to age-related cognitive deficits. Here, using specific antibodies against Ca_v_1.2 and Ca_v_1.3 subunits of LTCCs, we systematically re-examined the expression of these proteins in the hippocampus from young (3 to 4 month old) and aged (30 to 32 month old) F344xBN rats. Western blot analysis of the total expression levels revealed significant reductions in both Ca_v_1.2 and Ca_v_1.3 subunits from all three major hippocampal regions of aged rats. Despite the decreases in total expression levels, surface biotinylation experiments revealed significantly higher proportion of expression on the plasma membrane of Ca_v_1.2 in the CA1 and CA3 regions and of Ca_v_1.3 in the CA3 region from aged rats. Furthermore, the surface biotinylation results were supported by immunohistochemical analysis that revealed significant increases in Ca_v_1.2 immunoreactivity in the CA1 and CA3 regions of aged hippocampal pyramidal neurons. In addition, we found a significant increase in the level of phosphorylated Ca_v_1.2 on the plasma membrane in the dentate gyrus of aged rats. Taken together, our present findings strongly suggest that age-related cognitive deficits cannot be attributed to a global change in L-type channel expression nor to the level of phosphorylation of Ca_v_1.2 on the plasma membrane of hippocampal neurons. Rather, increased expression and density of LTCCs on the plasma membrane may underlie the age-related increase in L-type Ca^2+^ channel activity in CA1 pyramidal neurons.

## Introduction

The calcium hypothesis of aging (Khachaturian, 1987; Landfield, 1987) posits that age-related cognitive deficits are mainly due to changes in cellular mechanisms that maintain and regulate intracellular Ca^2+^ homeostasis. Among them, change in Ca^2+^ channel number and/or function has been suggested to be a key factor (Khachaturian, 1994). While age-related increase in function of L-type Ca^2+^ channels (LTCCs) in CA1 pyramidal neurons (Moyer & Disterhoft, 1994; Thibault & Landfield, 1996) and rescue of normal aging- and Alzheimer’s disease-related cognitive deficits with LTCC antagonists have been demonstrated (Deyo *et al*., 1989; Ban *et al*., 1990), there is conflicting evidence for altered number of LTCCs with aging. Increased (Herman *et al*., 1998; Chen *et al*., 2000; Veng & Browning, 2002), no change (Blalock *et al*., 2003; Kadish *et al*., 2009), and reduced (Rowe *et al*., 2007) expression levels of the central pore-forming α_1_-subunits of the L-type Ca^2+^ channels Ca_v_1.2 and Ca_v_1.3 in hippocampus from aged animals have been reported. These apparently conflicting findings may be due to the level of analysis conducted: from single cell to whole hippocampus. Therefore, we systematically examined the expression levels of Ca_v_1.2 and Ca_v_1.3 using Western blot, immunohistochemistry, and real-time quantitative PCR analysis in the three major hippocampal regions of young and aged rats.

## Results

### Ca_v_1.2 and Ca_v_1.3 protein levels are reduced in the aged hippocampus

**Figure 1:**
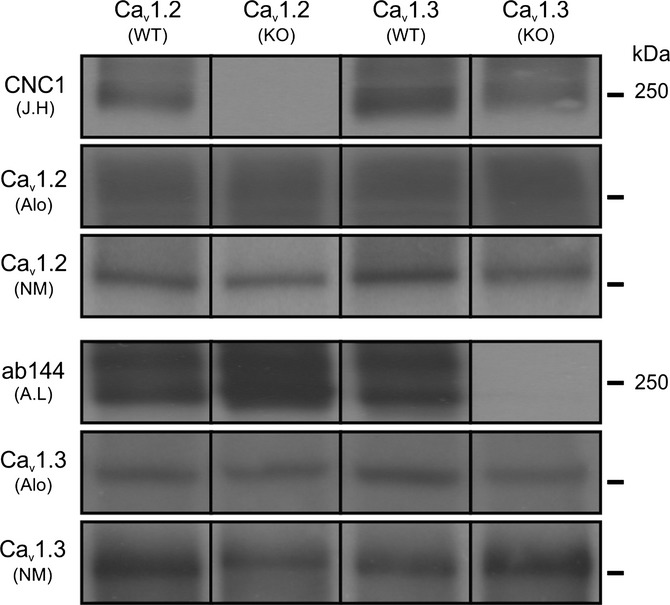
Characterization of antibody specificity for Ca_v_1.2 and Ca_v_1.3 proteins. Hippocampal lysates from wild-type (WT) and L-type-deficient (KO) mice were resolved by SDS-PAGE and immunoblotted with either CNC1 (J.H: Johannes W. Hell), ab144 (A.L: Amy Lee), commercially available anti-Ca_v_1.2 (Alo: Alomone Labs, ACC-003; NM: Neuromab Antibodies Inc. L57/46,) or commercially available anti-Ca_v_1.3 (Alo: Alomone Labs, ACC-005; NM: Neuromab Antibodies Inc. N38/8) antibodies. Blots were developed using Amersham ECL Plus and Hyperfilm ECL. Both anti-Ca_v_1.2 and anti-Ca_v_1.3 antibodies from commercial sources revealed nonspecific bands in hippocampal lysates from KO tissue. CNC1 and ab144 showed no cross-reactivity with either Ca_v_1.3 or Ca_v_1.2 proteins in hippocampal lysates. Note that this example figure is assembled from multiple blots with similar exposure time that have been aligned for illustrative purposes only. See Fig. S1 for immunoblots as loaded in gel.

**Figure 2:**
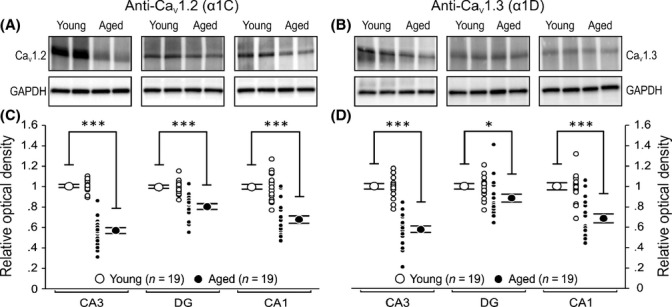
Total Ca_v_1.2 and Ca_v_1.3 L-type calcium channel protein levels are reduced in all three major hippocampal regions of aged rats. Homogenates from whole CA3, DG, and CA1 of dorsal hippocampus (four 1-mm-thick slices per animal) were analyzed using semi-quantitative Western blotting techniques and immunoblotted using highly specific antibodies against Ca_v_1.2 and Ca_v_1.3 L-type calcium channel α_1_ subunits. (A, B) Representative Western blots comparing expression of Ca_v_1.2 and Ca_v_1.3 proteins in CA3, DG, and CA1 regions from two young and two aged rats. Young and aged CA3, DG, and CA1 region samples were resolved in pairs (side by side) on the same gel. Note that a shorter exposure time was used for DG region for the purpose of illustration (See Figs S2 and S5). (C, D) Quantitation of total L-type calcium channel expression normalized to GAPDH and relative to young for each region. All results were confirmed by repeating the experiments and analysis three times. Significant reductions in Ca_v_1.2 and Ca_v_1.3 were observed in all three major hippocampal regions of aged animals. Unpaired *t*-test: *P < 0.05, ***P < 0.0001. Data reported as the mean ± SEM.

Ca_v_1.2 and Ca_v_1.3 expression levels were examined in CA1, CA3, and dentate gyrus (DG) of young (*N* = 19) and aged (*N* = 19) rats using antibodies specific for the two α_1_ subunits of these LTCCs (Fig. 1, Fig. S1). We found significantly reduced expression of both Ca_v_1.2 and Ca_v_1.3 subunits in all three regions from aged rats (Fig. 2). Furthermore, the reductions were nearly identical for both subunits at each hippocampal region: 40% in CA3, 30% in CA1, and 10–20% in DG as compared with young adults (Fig. 2). Representative full-length blots from Western blot analyses are shown in Fig. S2.

This is the first demonstration that the protein levels of both LTCC α-subunits are reduced throughout the hippocampus of aged rats. However, this raised a conundrum: How can there be increased Ca^2+^ conductance via LTCCs in CA1 pyramidal neurons (Moyer & Disterhoft, 1994; Thibault & Landfield, 1996) with fewer pore-forming subunits? To address this question, we began by examining the level of the Ca_v_1.2 and Ca_v_1.3 subunits found on the plasma membrane.

### Surface/total ratios of Ca_v_1.2 and Ca_v_1.3 are increased in aged hippocampus

We postulated that the relative ratios of Ca_v_1.2 and/or Ca_v_1.3 detected on the surface of cell membranes might be elevated in hippocampal tissue from aged rats. To test this hypothesis, we performed cell surface biotinylation assays (Thomas-Crusells *et al*., 2003) on dorsal hippocampal slices from young (*N* = 9) and aged (*N* = 9) rats.

**Figure 3:**
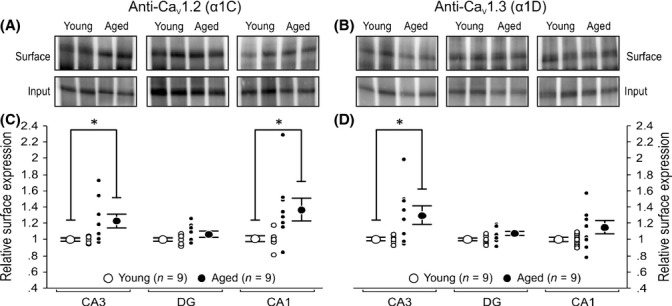
Age-related increases in the ratio of Ca_V_1.2 and Ca_V_1.3 proteins found on the plasma membrane as compared with the total protein levels found in specific regions of the hippocampus. Eight 250 μm thick acute hippocampal slices from 9 young and 9 aged rats were randomly selected and exposed to Sulfo-NHS–SS–biotin-labeling reagent before cell surface proteins were isolated using streptavidin magnetic beads. Control assays with biotinylated lysate proteins were also carried out to verify successful isolation of plasma membrane proteins in all three regions. We found little to no detection of our internal control protein, GAPDH, on surface fractions (See Fig. S3). (A, B) Representative Western blots comparing expression of Ca_v_1.2 and Ca_v_1.3 proteins in plasma membrane of CA3, DG, and CA1 from two young and two aged rats. Young and aged CA3, DG, and CA1 region samples were resolved in pairs (side by side) on the same gel. Note that a shorter exposure time was used for DG region for the purpose of illustration (See Figs S2 and S5). (C, D) Quantitation of surface L-type calcium channel protein expression normalized to young. (C) Ratio of surface-to-total expression of Ca_v_1.2 protein was found to be significantly higher in regions CA1 and CA3 of aged hippocampus. (D) Age-related increase in Ca_v_1.3 surface-to-total expression was only observed in the CA3 region. All results were confirmed by repeating the experiments and analysis twice. Unpaired *t*-test: *P < 0.05. Data reported as the mean ± SEM.

The surface/total ratio of Ca_v_1.2 subunit was significantly increased in CA1 (37%) and CA3 (22%) regions of aged rats (Fig. 3A,C). A similar surface/total ratio increase was also observed for the Ca_v_1.3 subunit, but it was significant only in the CA3 (29%) and not in the CA1 (15%) region of aged rats (Fig. 3B,D).

While these results demonstrate that higher levels of LTCCs are present on the cell membrane, they did not provide the location of the increased surface expression. Therefore, we performed immunohistochemical analysis to identify the locus of the increase.

### Ca_v_1.2 immunoreactivity (Ca_v_1.2-IR) is increased in somatic region of aged CA1 and CA3 neurons

CA1 pyramidal neurons from aged subjects have been shown to have increased LTCC activity (Thibault & Landfield, 1996) and enhanced calcium action potentials (Moyer & Disterhoft, 1994). Therefore, we postulated that increases in LTCC subunit expression would be observed mostly in the somatic region of aged CA1 pyramidal neurons.

**Figure 4:**
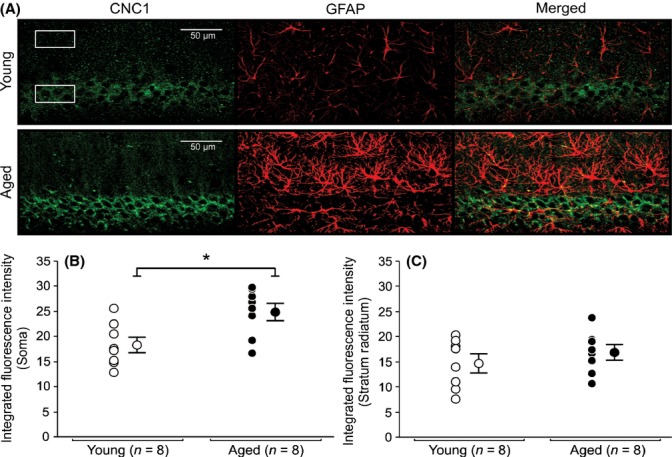
Expression of Ca_v_1.2 L-type subunit in soma and radiatum of young and aged CA1 pyramidal neurons. (A) Representative confocal images of hippocampal CA1 pyramidal layer sections showing immunohistochemical labeling for Ca_v_1.2 (CNC1) of a young and an aged rat. Six regions of interest (box) with equal dimensions in both the stratum pyramidale (3) and the stratum radiatum (3) layers of CA1 were drawn to collect immunofluorescence data. (B) Quantitative analysis of integrated fluorescence intensity in soma of CA1 pyramidal neurons of young and aged rats. (C) Quantitative analysis of integrated fluorescence intensity in the stratum radiatum of young and aged rats. Significant increases in somatic expression of Ca_v_1.2 subunit were observed in aged CA1 pyramidal neurons (B) (*P* < 0.05). No significant differences in Ca_v_1.2 subunit expression were detected between stratum radiatum of young and aged rats. No colocalization of CNC1 was observed in glial cells. AutoQuant image deconvolution software (Media Cybernetics, Rockville, MD) was used to reduce background signal for the purpose of illustration. Fluorescence intensities and analyses were performed using raw, unmodified, images. Data reported as the mean ± SEM.

**Figure 5:**
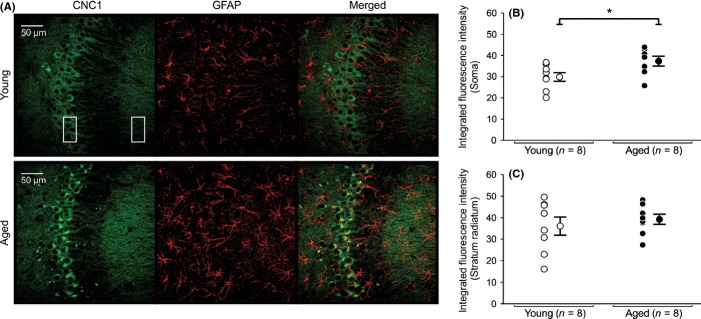
Expression of Ca_v_1.2 L-type subunit in soma and radiatum of young and aged CA3 pyramidal neurons. (A) Representative confocal images of hippocampal CA3 pyramidal layer sections showing immunohistochemical labeling for Ca_v_1.2 (CNC1) of a young and an aged rat. Six regions of interest (box) with equal dimensions in both the stratum pyramidale (3) and the stratum radiatum (3) layers of CA3 were drawn to collect immunofluorescence data. (B) Quantitative analysis of integrated fluorescence intensity in soma of CA3 pyramidal neurons of young and aged rats. (C) Quantitative analysis of integrated fluorescence intensity in the stratum radiatum of young and aged rats. Significant increases in somatic expression of Ca_v_1.2 subunit were observed in aged CA3 pyramidal neurons (B) (*P* < 0.05). No significant differences in Ca_v_1.2 subunit expression were detected between stratum radiatum of young and aged rats. No colocalization of CNC1 was observed in glial cells. AutoQuant image deconvolution software (Media Cybernetics, Rockville, MD) was used to reduce background signal for the purpose of illustration. Fluorescence intensities and analyses were performed using raw, unmodified, images. Data reported as the mean ± SEM.

**Figure 6:**
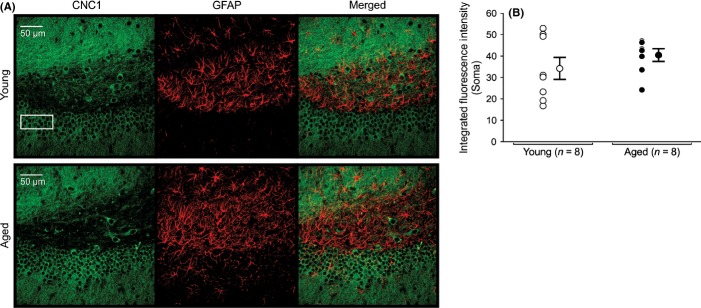
Expression of Ca_v_1.2 L-type subunit in DG granular cells of young and aged hippocampus. (A) Representative confocal images of hippocampal DG granular cells showing immunohistochemical labeling for Ca_v_1.2 (CNC1) of a young and an aged rat. Three regions of interest placed atop the granular layer were drawn to collect immunofluorescence data. (B) Quantitative analysis of integrated fluorescence intensity of granular cells in DG of young and aged rats. No significant differences in Ca_v_1.2 subunit expression were detected in granular cells of young and aged rats. No colocalization of CNC1 was observed in glial cells. AutoQuant image deconvolution software (Media Cybernetics, Rockville, MD) was used to reduce background signal for the purpose of illustration. Fluorescence intensities and analyses were performed using raw, unmodified, images. Data reported as the mean ± SEM.

We observed Ca_v_1.2-IR within the hippocampal formation and hippocampal cell layers similar to previous reports (Hell *et al*., 1993; Clark *et al*., 2003; Hall *et al*., 2013). Significant increases in Ca_v_1.2-IR were observed in the somatic regions of aged CA1 (Fig. 4) and CA3 (Fig. 5) pyramidal neurons. No change in Ca_v_1.2-IR and expression was observed in DG granule cells (Fig. 6). Furthermore, no significant changes in Ca_v_1.2 subunit expression were observed in stratum radiatum of CA1 (Fig. 4C) or CA3 (Fig. 5C) hippocampal region.

In addition, as LTCCs are found in glial cells and its expression has been documented to change with astrocyte activation after brain injury, trauma, and aging (Wisniewski & Terry, 1973; Vaughan & Peters, 1974; MacVicar, 1984; Westenbroek *et al*., 1998; Chung *et al*., 2001; Djamshidian *et al*., 2002; Finch, 2003; Xu *et al*., 2007), we also examined whether the observed plasma membrane increases in LTCC subunits with aging were of glial/astrocytic origin. No detectable expression/colocalization of LTCC subunits was observed in glial cells with our antibodies (Figs 4–6).

Parallel to Ca_v_1.2, immunohistochemical experiments were conducted to assess changes in Ca_v_1.3 protein expression at the cellular level. However, the presence of various nonspecific, high-intensity bands in our Ca_v_1.3 Western blots (Fig. S2B,D) prevents us from confidently reporting our Ca_v_1.3 immunohistochemical findings, as the obtained Ca_v_1.3 immunoreactivity might be the results of nonspecific binding of our current antibody in brain tissue. Hence, it remains to be determined whether immunohistochemical analyses of Ca_v_1.3 expression at the cellular level are consistent with our Ca_v_1.3 biotinylation experiments.

### Phosphorylation of surface-expressed Ca_v_1.2 is increased in the DG of aged rats

**Figure 7:**
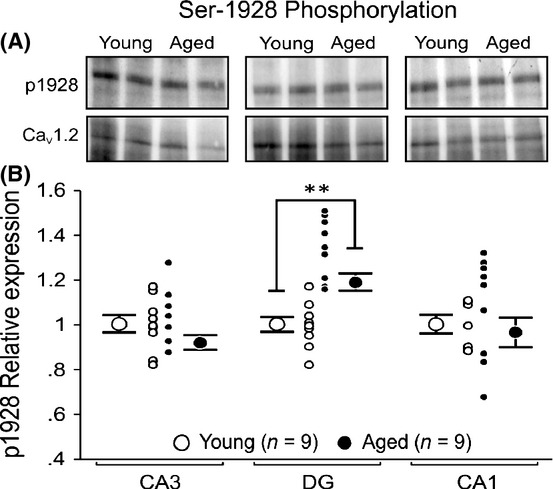
Phosphorylation of Serine 1928 is significantly increased in cell surface-expressed Ca_v_1.2 proteins in DG of aged rat. Biotinylated CA1, CA3, and DG cell surface fractions were resolved and immunoblotted with p1928 and CNC1 antibody to determine the relative expression of phosphorylated Ca_v_1.2 proteins localized at the cell membrane. The relative level of phosphorylation of Serine 1928 on Ca_v_1.2 protein was determined by normalizing the obtained p1928 signals to the corresponding CNC1 signals for the loaded surface fraction. (A) Representative Western blots comparing cell surface expression of p1928 and Ca_v_1.2 protein in CA3, DG, and CA1 from two young and two aged rats. (B) Quantitation of p1928 with relation to total cell surface-expressed Ca_v_1.2 protein in young and aged rats. We found no significant age-related differences in phosphorylation of Ca_V_1.2 on CA1 or CA3 region. However, a significant increase in phosphorylation was observed on DG region of aged rats. All results were confirmed by repeating the experiments and analysis twice. Unpaired *t*-test: **P < 0.005. Data reported as the mean ± SEM.

Increased LTCC activity by 4-6-fold has been reported when the Ca_v_1.2 subunits are phosphorylated (Sculptoreanu *et al*., 1993; Kavalali *et al*., 1997). In addition, Serine 1928 (S1928) (Davare & Hell, 2003) and Serine 1700 (S1700) (Fuller *et al*., 2010) of Ca_v_1.2 can be phosphorylated, but only S1928 phosphorylation has been shown to be increased with normal aging (Davare & Hell, 2003). Therefore, we further explored the possibility that more S1928 in Ca_v_1.2 might be phosphorylated in the hippocampal regions from aged rats. Biotinylated plasma membrane proteins were isolated and immunoblotted with anti-CH1923-1932P (p1928), an antibody designed to specifically detect Ca_v_1.2 when phosphorylated at S1928 (De Jongh *et al*., 1996; Davare & Hell, 2003). We found a significant 1.18-fold increase in phosphorylated Ca_v_1.2 in DG of aged rats (Fig. 7B). No significant age-related difference in phosphorylated Ca_v_1.2 was detected in either CA1 or CA3.

### Ca_v_1.2 and Ca_v_1.3 mRNA levels are unchanged with aging

A positive correlation between Ca_v_1.3 mRNA and LTCC activity has been previously demonstrated in CA1 pyramidal neurons using single cell reverse transcription PCR experiments (Chen *et al*., 2000). Therefore, we performed a systematic assay to assess Ca_v_1.2 and Ca_v_1.3 mRNA levels in each hippocampal region using the real-time quantitative PCR method to determine whether the mRNA levels were altered with normal aging and/or in a specific hippocampal region(s). We found no significant age-related changes in mRNA levels for both Ca_v_1.2 and Ca_v_1.3 (Table 1, Fig. S4).

**Table 1:** L-type calcium channel mRNA levels within the hippocampal subregions are not altered by normal aging

	CA1		CA3		DG	
	▵*C*_T_^YOUNG^	▵*C*_T_^OLD^	▵*C*_T_^YOUNG^	▵*C*_T_^OLD^	▵*C*_T_^YOUNG^	▵*C*_T_^OLD^
Ca_v_1.2 RT-PCR						
Pair 1	5.66	5.66	4.94	5.15	6.72	7.02
Pair 2	5.68	5.30	5.04	5.10	7.15	6.96
Pair 3	5.54	5.34	4.79	4.68	6.68	7.02
Pair 4	5.44	5.73	4.86	5.20	7.05	7.27
Pair 5	5.85	5.52	5.02	4.90	6.92	6.73
Pair 6	5.69	5.90	4.95	4.73	7.03	6.80
Pair 7	5.64	5.38	4.84	4.69	6.78	6.92
Average ± SD	5.64 ± 0.13	5.55 ± 0.22	4.92 ± 0.09	4.92 ± 0.23	6.90 ± 0.18	6.96 ± 0.17
▵▵*C*_T_ (▵*C*_T_^OLD^ – ▵*C*_T_^YOUNG^)		−0.10 ± 0.26		0.00 ± 0.25		0.06 ± 0.25
Fold change (2^−▵▵*C*^T)		1.07		1.00		0.96
Ca_v_1.3 RT-PCR						
Pair 1	7.29	7.30	5.78	5.91	6.76	7.19
Pair 2	7.10	6.96	5.78	5.77	7.14	7.44
Pair 3	7.13	7.12	5.54	5.59	7.10	7.49
Pair 4	6.85	7.25	5.59	5.91	7.24	7.19
Pair 5	7.30	7.05	5.89	5.75	7.06	6.96
Pair 6	7.42	7.30	5.56	5.57	7.22	7.20
Pair 7	7.06	6.78	5.63	5.53	7.32	7.39
Average ± SD	7.16 ± 0.19	7.11 ± 0.19	5.68 ± 0.13	5.72 ± 0.16	7.12 ± 0.18	7.27 ± 0.18
▵▵*C*_T_ (▵*C*_T_^OLD^ – ▵*C*_T_^Y0UNG^)		−0.05 ± 0.27		0.04 ± 0.21		0.15 ± 0.26
Fold change (2^−▵▵*C*^T)		1.04		0.98		0.96

Total RNA was isolated from major hippocampal regions of young and aged rats. Equivalent amounts were converted into cDNA. Real-time quantitative PCR was performed on triplicates of subject for Ca_v_1.2, Ca_v_1.3, and GAPDH. Δ*C*_T_ is calculated by subtracting threshold fluorescence of internal housekeeping gene GAPDH, for example, (*C*_T_^CaV1.2^ – *C*_T_^Gapdh^).

## Discussion

The present study is the first to demonstrate that the pore-forming α_1_ subunits for the L-type voltage-gated Ca^2+^ channels (Ca_v_1.2 and Ca_v_1.3) are significantly reduced in whole tissue lysates from all three major hippocampal regions of aged rats (Fig. 2). However, the biotinylation and immunohistochemical data demonstrate that age-related increases in Ca_v_1.2 are observed in CA1 and CA3 regions. Notably, the increase in the Ca_v_1.2 subunit was in the somatic region of CA1 and CA3 pyramidal neurons (Figs 4 and 5). In addition, no detectable expression/colocalization of Ca_v_1.2 subunits was observed in glial cells with our antibodies (Figs 4–6). Therefore, the present results support the ‘calcium hypothesis of aging’ (Khachaturian, 1987; Landfield, 1987) in that the age-related increase in surface expression of L-type voltage-gated Ca^2+^ channels (Ca_v_1.2 and Ca_v_1.3) in hippocampal pyramidal neurons we demonstrate may play an important role in the cognitive deficits observed in normal aging subjects.

Phosphorylation of Ca_v_1.2 LTCC has been previously shown to enhance Ca^2+^ influx (Sculptoreanu *et al*., 1993; Kavalali *et al*., 1997). Ca_v_1.2 α_1_ subunit can be phosphorylated at Serine 1928 (S1928) (Davare & Hell, 2003) and at Serine 1700 (S1700) (Fuller *et al*., 2010), and it has been suggested that S1700 phosphorylation plays a greater modulatory role than S1928 phosphorylation (Brandmayr *et al*., 2012). However, only S1928 phosphorylation has been shown to be increased in the hippocampus with normal aging (Davare & Hell, 2003). Similarly, we also found significant age-related increase in S1928 phosphorylation, but only in the DG with no apparent changes in other hippocampal regions (Fig. 7). The difference with the previous report (Davare & Hell, 2003) may be due to our focus on the phosphorylation of cell surface channels in each hippocampal region of the dorsal hippocampus, whereas the previous report examined S1928 phosphorylation in the entire hippocampus. In addition, we found high level of Ca_v_1.2 protein in the DG as compared with CA1 and CA3 from whole tissue lysate (Fig. S5). Furthermore, no age-related changes in the Ca^2^^+^ -dependent postburst afterhyperpolarization have been observed in DG granule cells (Baskys *et al*., 1987; Niesen *et al*., 1988; Reynolds & Carlen, 1989). Therefore, S1928 phosphorylation cannot account for increased calcium influx and reduced neuronal excitability with normal aging observed in CA1 hippocampal pyramidal neurons (Landfield & Pitler, 1984; Moyer *et al*., 1992; Moyer & Disterhoft, 1994; Thibault & Landfield, 1996).

We found no age-related changes in Ca_v_1.2 or Ca_v_1.3 mRNA expression in the present study. Previous reports examining the mRNA or LTCC α-subunits in hippocampus from aged animals have been inconsistent; with groups reporting an increase (Herman *et al*., 1998; Chen *et al*., 2000; Veng & Browning, 2002), no change (Blalock *et al*., 2003; Kadish *et al*., 2009), or reductions (Rowe *et al*., 2007) in mRNA or α-subunit expression with aging. The discrepancy between the findings may be due to the anatomical specificity and/or method of analysis used in the studies (i.e. differences in splice variants amplified by the different primers of different groups). At single cell resolution, a positive correlation between Ca_v_1.3 mRNA levels and functional channel density in the adult and aged CA1 pyramidal neurons has been reported: that is, the more Ca_v_1.3 mRNA, the greater the LTCC activity (Chen *et al*., 2000). Using whole hippocampus, a report using semi-quantitative RNAse protection analysis revealed that Ca_v_1.2 and Ca_v_1.3 mRNA expression levels are increased in aged rats (Herman *et al*., 1998), whereas no change in either mRNA (Blalock *et al*., 2003; Kadish *et al*., 2009) or a reduction in Ca_v_1.2 mRNA (Rowe *et al*., 2007) were reported using microarray methods. Our present real-time quantitative PCR data support the lack of change in Ca_v_1.2 or Ca_v_1.3 mRNA expression level with normal aging in the subregions of the hippocampus (Table 1). Notably, we also observed that the level of Ca_v_1.2 and Ca_v_1.3 mRNA expressions was different than that for the protein levels: for protein, DG > CA3 ≥ CA1 (Fig. S5): for mRNA, CA3 ≥ CA1 > DG (Table 1). Furthermore, a 4-fold increase in Ca_v_1.2 mRNA relative to Ca_v_1.3 mRNA was observed in the CA1 region, which supports the previous reports that approximately 80% of LTCCs are Ca_v_1.2 channels (Hell *et al*., 1993; Clark *et al*., 2003).

The exact mechanism by which LTCC activity is increased with aging in CA1 neurons is as yet unclear. While our findings provide a better understanding of the processes that take place during aging, we cannot exclude the role that other processes may have in channel function, activity, and intracellular Ca^2+^ concentrations. For example, the activity of the pore-forming LTCC α-subunits can be regulated by protein–protein interactions, a number of which can enhance L-type channel function (Catterall, 2000; Calin-Jageman & Lee, 2008). Second, calcineurin expression levels and activity have also been shown to be increased in hippocampus from aged animals (Foster *et al*., 2001; Norris *et al*., 2005; Eto *et al*., 2008) and inhibiting it reduces LTCC activity (Norris *et al*., 2002, 2010) and the Ca^2+^-dependent postburst afterhyperpolarization (AHP) (Vogalis *et al*., 2004). Third, post-translational modifications of the Ca_v_1.2 LTCC protein by proteolytic cleavage of the C-terminus region can significantly impact voltage-dependent activation and activity of the channel (Wei *et al*., 1994; Hulme *et al*., 2006). Finally, Ca_v_1.2 and Ca_v_1.3 channels are regulated by proteosomal degradation (Altier *et al*., 2011; Gregory *et al*., 2011) and oxidative stress through the action of reactive oxygen species, which can lead to increased accumulation of Ca^2+^ inside hippocampal neurons. (Kourie, 1998; Fusi *et al*., 2001).

Age-related increase in LTCC function, specifically in CA1 pyramidal neurons, has been a popular hypothesis to explain age-related cognitive deficits (Disterhoft & Oh, 2006; Foster, 2007; Thibault *et al*., 2007). Previous reports have demonstrated that Ca^2+^ influx into neurons is significantly increased in CA1 pyramidal neurons from aged animals (Moyer & Disterhoft, 1994; Thibault & Landfield, 1996) due to increased density of LTCCs (Thibault & Landfield, 1996), which leads to reduced intrinsic excitability (Landfield & Pitler, 1984; Thompson *et al*., 1990; Moyer *et al*., 1992) and synaptic plasticity (Norris *et al*., 1998). Rescue of age-related cognitive deficits (Deyo *et al*., 1989) and restoration of intrinsic neuronal excitability (Moyer *et al*., 1992; Norris *et al*., 1998) and synaptic plasticity (Norris *et al*., 1998) with the use of LTCC blockers (e.g. nimodipine and nifedipine) have further provided support for the previously held viewpoint. In addition to the enhanced Ca^2+^ influx via LTCCs, Ca^2+^ released from the endoplasmic reticulum through ryanodine receptors via the Ca^2+^-induced Ca^2+^-release (CICR) mechanism has been suggested and shown to greatly reduce excitability (i.e., increase postburst AHP) of CA1 pyramidal neurons from aged rats (Kumar & Foster, 2004; Gant *et al*., 2006; Kim *et al*., 2007; Thibault *et al*., 2007). Therefore, while increased LTCC function is a significant source of the age-related cognitive deficits, other sources of Ca^2+^ that are changed with normal aging should also be systematically characterized.

The increased LTCC surface expression was not observed in all of the aged rats examined (Figs 3–5). This heterogeneity was expected as we have previously reported that nearly half of the aged cohort of 27–31-month-old F344xBN rats are learning-impaired (Knuttinen *et al*., 2001; Matthews *et al*., 2009). Similar age-related heterogeneity has been reported for other strains of rats (Gallagher *et al*., 1993; Tombaugh *et al*., 2005) and in rabbits (Thompson *et al*., 1996; Moyer *et al*., 2000). More importantly, it has been demonstrated that learning-impaired aged animals have CA1 pyramidal neurons with enlarged postburst AHP (Moyer *et al*., 2000; Tombaugh *et al*., 2005; Matthews *et al*., 2009). Therefore, we hypothesize that only those aged subjects with increased surface expression of functional LTCCs are likely to be cognitively impaired given the contribution of Ca^2+^ influx via L-type Ca^2+^ channels to the AHP. Additional studies are required to determine whether such correlation exists.

In summary, our results suggest that age-related cognitive deficits cannot be attributed to a global change in L-type Ca^2+^ channel expression or to the level of phosphorylation of Ca_v_1.2 channels in the plasma membrane of aged hippocampal neurons. Rather, we provide evidence that age-related increases in plasma membrane expression and/or distribution of L-type Ca^2+^ channels in the somatic regions of CA1 and CA3 pyramidal neurons may underlie the reported changes in neuronal excitability and activity observed with normal aging.

## Materials and methods

### Subjects

Young adults (3–4 month old) and aged (30–32 month old) male F1 hybrid Fischer 344 X Brown Norway rats (F344xBN; Harlan, Indianapolis, IN, USA) were used in this study. Rats were group housed with ad libitum access to food and water and maintained in a climate-controlled room with a 14:10 h light/dark cycle. The F344xBN rats are long-lived with > 50% survival rate at 34 months of age (National Institute on Aging, Aged Rodent Colonies Handbook, www.nia.nih.gov/research/dab/aged-rodent-colonies-handbook/ strain-survival-information) and have significantly less pathological complications with normal aging as compared with the Fischer 344 (F344) rats (Bronson, 1990; Lipman *et al*., 1996). Every effort was made to ensure that only healthy rats were included in the experiments. Rats with palpable tumors, skin ulcerations, infections, or difficulty moving were excluded from the studies. All experimental procedures were approved by the Northwestern University Animal Care and Use Committee and conformed to NIH standards (NIH Publications No. 80-23). All efforts were made to minimize animals’ discomfort and the number of animals used.

For CNC1 antibody specificity, brains and cerebellar tissue from conditional knockout mice with a forebrain-specific (hippocampus and forebrain) deletion of Ca_v_1.2 were used (McKinney *et al*., 2008; White *et al*., 2008). For ab144 antibody specificity, brains from knockout mice with a global deletion of the gene encoding Ca_v_1.3 were used (Platzer *et al*., 2000; Clark *et al*., 2003; McKinney & Murphy, 2006). Tissue from knockout mice and their wild-type littermates were generously provided by Dr. Geoffrey G. Murphy (University of Michigan, Ann Arbor, MI, USA).

### Antibodies

The previously characterized rabbit anti-Ca_v_1.2 (CNC1) antibody (provided by J.W. Hell) was raised against a peptide covering residues 818 – 835 within the cytoplasmic loop between domains II and III of the Ca_v_1.2 protein (Hell *et al*., 1993; Hall *et al*., 2013). The rabbit anti-Ca_v_1.3 (Ab144) antibody (provided by A. Lee) was raised against a synthetic peptide corresponding to Ca_v_1.3 N-terminal sequence (MQHQRQQQEDHANEANYARGTRKC; Covance Research Products, Denver, PA, USA) (Jenkins *et al*., 2010; Gregory *et al*., 2011). Specificity is further demonstrated in Fig. S1. The rabbit anti-CH1923-1932P (p1928) antibody, which specifically binds to phosphorylated Ca_v_1.2 when phosphorylated at Serine 1928, was raised against a phosphopeptide consisting of residues 1923–1932 (De Jongh *et al*., 1996; Davare & Hell, 2003). Rabbit polyclonal antiglyceraldehyde-3-phosphate dehydrogenase (GAPDH) antibody was obtained from Abcam (Cambridge, MA, USA) and chicken anti-glial fibrillary acidic protein (GFAP) antibody was obtained from Millipore (Temecula, CA, USA).

### Sample preparation and Immunoblotting

Whole dorsal hippocampi from 19 young and 19 aged rats were dissected out and immediately placed in cold (approximately 0 °C) oxygenated (95%/5% O_2_/CO_2_) aCSF (in mm: 124 NaCl, 1.25 NaH_2_PO_4_, 2.5 KCl, 26 NaHCO_3_, 25 glucose, 2.4 CaCl_2_, and 2.0 MgSO_4_, pH 7.4) before transverse dorsal hippocampal slices (400 μm) were made using a manual tissue slicer (Stoelting Co., Wood Dale, IL, USA). The hippocampal slices were then transferred to fresh oxygenated ice-cold aCSF containing several protease and phosphatase inhibitors (1 μg mL^−1^ pepstatin A, 10 μg mL^−1^ leupeptin, 20 μg mL^−1^ aprotinin, 200 nm phenylmethanesulfonyl fluoride, 8 μg mL^−1^ calpain inhibitor I, 8 μg mL^−1^ calpain inhibitor II, 1 mm *p*-nitrophenyl phosphate, 50 mm NaF, 20 mm sodium pyrophosphate, and 4 μm microcystin LR) and immediately microdissected under a microscope (Zeiss Stemi DV4) to yield the three major hippocampal subdivisions (CA1, CA3, and Dentate Gyrus) (Coultrap *et al*., 2005). Each microdissected region was individually homogenized and sonicated in 1% Triton X-100 lysis buffer containing protease and phosphatase inhibitors and cleared by ultracentrifugation. Protein concentration was determined by the BCA assay using bovine serum albumin (BSA) as a standard (Pierce, Rockford, IL, USA).

For quantification of total L-type Ca^2+^ channel expression in the three major hippocampal regions of young and aged rats, samples containing equal amounts of proteins (20 μg) were resolved by SDS-PAGE and analyzed by immunoblotting with either anti-CNC1 or Ab144. Fresh blots were used for each channel of interest, and each blot was reprobed with GAPDH for normalization and to control for variability during sample loading. Immunoreactive bands were visualized using a ChemiDoc XRS+ Molecular Imager System with Image Lab™ Software (Bio-Rad Laboratories, Hercules, CA, USA), and only signals doubling with increasing exposure times were used for quantification and analysis. All immunoblots were measured and quantified by densitometry using NIH imagej image analysis software (rsb.info.nih.gov/ij/).

### Whole slice cell surface biotinylation

To assess the relative cell surface expression of L-type Ca^2+^ channels in hippocampal pyramidal neurons, cell surface biotinylation experiments were conducted on acute hippocampal slices (*n* = 8, 250 μm slices per rat) from nine young and nine aged rats. This technique has been successfully shown to reach all layers of acute hippocampal slices of up to 400 μm in thickness using Sulfo-NHS–SS–biotin as labeling reagent with very low to no labeling of intracellular proteins (Thomas-Crusells *et al*., 2003). Alternate slices from the dorsal half of both left and right hippocampi were exposed to 1 mg mL^−1^ Sulfo-NHS–SS–bio*t*in-labeling reagent (Pierce) for 30 min before separating each hippocampal region for processing and isolation of surface proteins using streptavidin magnetic beads (Pierce).

Immunoblotting with p1928, CNC1, and Ab144 antibody was performed after SDS-PAGE separation of total region lysates (input) and biotinylated (surface) fraction proteins. GAPDH was used as both loading and internal protein control to confirm the success of the biotinylation procedures (Fig. S3).

### L-type Ca^2+^ channel surface expression and phosphorylation of Ca_v_1.2 measurements

Following Western blot analysis, optical density values for total lysate (input) and biotinylated (surface) fractions were obtained and the relative ratio of surface expression for each subunit was determined by normalizing the surface optical density value to its corresponding input band density value. The level of phosphorylation of Serine 1928 on Ca_v_1.2 protein was determined by normalizing the obtained p1928 band to the total CNC1 intensity for the loaded surface fraction. To quantify the level of p1928 phosphorylation of surface-expressed Ca_v_1.2 channels, blots were initially probed with p1928, stripped, and reprobed with CNC1 antibody.

### RNA isolation and cDNA synthesis

CA1, CA3, and DG regions were isolated from young and aged rats in pairs, homogenized in RPLT-Plus Lysis Buffer (Qiagen, Valencia, CA, USA) and stored at −80 °C until RNA isolation. Samples were further dissociated with QiaShredder columns, and the total RNA was isolated via Qiagen RNEasy Plus Kit according to manufacturer’s directions. RNA was dissolved into 60 μl RNAse-free water, stored on ice, and the yield was determined with a nanodrop spectrophotometer (Thermo-Scientific, Rockford, IL, USA). 650 ng (CA1, CA3) or 240 ng (DG) of total RNA was converted into cDNA with reverse transcriptase and multiple primers (qScript™ cDNA SuperMix; Quanta Biosciences, Gaithersburg, MD, USA) on a thermocycler per manufacturer’s instructions and stored at −20 °C until use. Synthesis of cDNA was commenced within 2 h of RNA isolation.

### Immunohistochemistry

Young (3–4 month old) and aged (30–32 month old) male F344xBN rats were anesthetized and intracardially perfused with ice-cold sodium phosphate buffer (0.1 m PB [pH 7.4]) supplemented with several protease and phosphatase inhibitors (PPI; cOmplete and PhosSTOP Inhibitor Cocktail Tablets; Roche, Indianapolis, IN, USA) and followed by ice-cold 4% paraformaldehyde in PB (supplemented with PPI). Brains were removed, postfixed (overnight), and cryoprotected by successively sinking in 10% (w/v) and 30% (w/v) sucrose in PB at 4 °C for 72 h (Marshall *et al*., 2011). Forty-micrometer-thick coronal sections containing the hippocampus were made, hippocampus dissected out, and stored at −20 °C in cryoprotectant solution (0.1 m PBS, pH 7.4, 30% (w/v) sucrose, 30% (v/v) ethylene glycol and 1% (w/v) polyvinylpyrrolidone) (Watson *et al*., 1986) until processed for immunohistochemistry.

Immunohistochemistry (IHC) was performed as previously described (Ferraguti *et al*., 2004; Wu *et al*., 2008) with some modifications. The tissue processing and data collection and analysis were performed blind to the age of the animals. Five hippocampal slices from each animal were systemically and randomly selected for double immunolabeling with antibodies against glial fibrillary acidic protein (GFAP) and Ca_v_1.2 or Ca_v_1.3. The free-floating sections were rinsed in 0.1 m Dulbecco’s phosphate-buffered saline (DPBS) for 30 min to remove cryoprotectant, incubated with 0.05% boric acid in DPBS for 10 min, rinsed in DPBS containing 0.1% Tween-20 (DPBS-T) for 30 min, and blocked in DPBS containing 5% normal donkey serum, 1% immunoglobulin- and protease**-**free bovine serum albumin, and 0.3% Triton X-100 for 2 h. The sections were then incubated in chicken anti-GFAP (diluted 1:500) and anti-Ca_v_1.2 (CNC1; diluted 1:500) or anti-Ca_v_1.3 (ab144; diluted 1:2000) overnight at 4 °C, rinsed in DPBS-T for 30 min, incubated in FITC-conjugated secondary antibody against rabbit IgG (diluted 1:500) and Texas Red-conjugated secondary antibody against chicken IgG (diluted 1:500) (Jackson immunoresearch, West Grove, PA) for 1 h at room temperature, rinsed in DPBS-T for 1 h, rinsed in DPBS for 10 min, and finally mounted and coverslipped using ProLong® Gold antifade reagent with DAPI (Molecular Probes, Eugene, OR, USA). Control hippocampal sections from young and aged rats were also included in the assay and treated the same way, but either the primary, secondary, or both antibodies were excluded from the incubating solution. All slices from young and aged animals were simultaneously processed during the experiment in order to minimize the effects of potential inter-batch staining variability.

### Image analysis

Confocal images from CA1 region of hippocampus were obtained at a magnification of 40× using a Nikon Eclipse C1si Spectral Imaging Confocal Microscope System at the Nikon Imaging Center and Cell Imaging Facility at Northwestern University. Exposure parameters for Ca_v_1.2, Ca_v_1.3, and GFAP were standardized across all captured images and maintained throughout image acquisition for both young and aged hippocampal slides. Images were analyzed using MetaMorph® imaging software (Molecular Devices, Sunnyvale, CA, USA), and statistical analyses were performed using StatView software. To study age-related changes in L-type subunit expression in CA1 and CA3, data were collected from raw, unmodified, images by drawing three rectangular regions of interest (ROI) with equal dimensions in both the stratum pyramidale and the stratum radiatum layers of CA1 and CA3 regions. For DG, ROIs were placed atop the granular cell layer. The fluorescence intensity for each ROI was then averaged to calculate the integrated fluorescent intensity for each hippocampal slice. Averaged values from all 5 hippocampal slices from each animal were then averaged to collect the animal’s integrated fluorescent intensity used for plotting and statistical analysis. Significant group differences in protein expression were evaluated using analysis of variance (ANOVA) with statistical significance set to *P* < 0.05. All data are reported as the means ± SEM.

### Real-time PCR of mRNA levels

Primer sequences compatible with rat and mouse were a gift from C. Savio Chan (Northwestern University). The primers were designed to bridge exons of cDNA to eliminate concern of genomic DNA contamination and previously tested for comparable efficiency during PCR. Primers include Cacna1c, bridging exons 7–8 [sense primer-GGCATCACCAACTTCGACA, antisense Primer- TACACCCAGGGCAACTCATA], Cacna1d, bridging exons 41–42 [sense primer-TGACATTGGGCCAGAAATCC, antisense primer- GGTGGTATTGGTCTGCTGAA], and GAPDH, bridging exons 3–4 [sense primer- GCTGAGTATGTCGTGGAGTCTA, antisense primer- TTCTCGTGGTTCACACCCAT]. For real-time quantitative PCR, 1 pair of young and aged cDNA (1 μL) triplicate samples from each of CA1, CA3, and DG were measured in parallel for threshold fluorescence accumulation (*C*_T_) of each gene target (Cacna1c, Cacna1d, and GAPDH) in a 96-well tray with a Step One Plus™ Applied Biosystems QPCR Machine using SYBR Green as a reporter and ROX™ dye as a passive reference control. After PCR, a melt curve analysis was done for each sample to ensure that the primers were specific. The threshold *C*_T_ was manually set to be 1.1 Δ*R*n (reporter-reference control baseline fluorescence) for all targets and fell within the exponential phase of amplification. The comparative ΔΔ*C*_T_ method described by Livak and Schmittgen (Livak & Schmittgen, 2001) was used to compare young and aged samples with GAPDH, which was used as internal housekeeping control. Δ*C*_T_ for each sample target gene was calculated as follows: Δ*C*_T_ = (mean *C*_T_^Target^ – mean *C*_T_^Gapdh^). To obtain fold change due to age, ΔΔ*C*_T_ was calculated with the following equation: (Δ*C*_T_^AGED^ – Δ*C*_T_^Young^) and presented in Table 1.

### Statistical analysis

All statistical analyses were performed using statview analysis software, and significant group differences in protein expression were evaluated using analysis of variance (ANOVA) with statistical significance set to *P* < 0.05. All data are reported as the mean ± SEM. Duplicates were performed on all reported phosphorylation and surface expression assays.

## Supporting Information

**Fig. S1** Depiction of Immunoblots from CNC1 and ab144 antibody specificity on hippocampal and cerebellar tissue lysates.

**Fig. S2** Examples depicting full length Immunoblots from Western blot analyses.

**Fig. S3** Selective isolation of surface-expressed proteins.

**Fig. S4** Examples depicting qRT-PCR amplification plots for Ca_v_1.2 and Ca_v_1.3 gene expression analyses.

**Fig. S5** Regional expression of Ca_v_1.2 and Ca_v_1.3 L-type calcium channel proteins in dorsal hippocampus.
