# Supplementary material for: Surface L-type Ca2+ channel expression levels are increased in aged hippocampus
Source: Aging Cell. 2013 Oct 1;13(1):111–20. doi: 10.1111/acel.12157 (PMC3947046; doi:10.1111/acel.12157)
Supplement: Supplementary file 6 [file acel0013-0111-sd6.docx]

**SUPPLEMENTAL FIGURE LEGENDS:**

**Figure S1. Depiction of Immunoblots from CNC1 and ab144 antibody specificity on hippocampal and cerebellar tissue lysates.** Dorsal hippocampal (HC) and cerebellar (Crb) lysates from wild-type (WT) and knockout (KO) mice were resolved by SDS-PAGE and immunoblotted with CNC1 (Ca_v_1.2 J.H: Johannes W. Hell), ab144 (Ca_v_1.3 A.L: Amy Lee), Alo: Alomone Labs (anti-Ca_v_1.2, ACC-003; anti-Ca_v_1.3, ACC-005), NM: Neuromab Antibodies Inc. (anti-Ca_v_1.2, L57/46; anti-Ca_v_1.3, N38/8) antibody. Blots were developed using Amersham ECL Plus and Hyperfilm ECL. As expected, CNC1 antibody labeled proteins consistent in size with Ca_v_1.2 in both rat and Ca_v_1.2-WT mice but not in Ca_v_1.2-KO mice (A, C). The ab144 antibody detected both a >250 kDa protein and a >170 kDa protein in hippocampal lysates from Ca_v_1.3-WT mice but not Ca_v_1.3-KO mice (B, D). Both commercial anti-Ca_v_1.2 and anti-Ca_v_1.3 antibodies used during our specificity tests showed nonspecific immunoreactivity in hippocampal and cerebellar lysates from KO tissue (A, B, C, D, lower panels). Wild-type and knockout mice tissue were generously provided by Dr. Geoffrey G. Murphy, University of Michigan, Ann Arbor. MI.

**Figure S2. Examples depicting full length Immunoblots from western analyses.** KO: Knockout, CNC1 (A, C): anti-Ca_v_1.2 (Johannes W. Hell), Ab144 (B, D): anti-Ca_v_1.3 (Amy Lee). GAPDH: antiglyceraldehyde 3-phosphate dehydrogenase (Abcam). GAPDH was used for both data normalization and as loading control.

**Figure S3. Selective isolation of surface-expressed proteins.** Biotinylated proteins from each of the three major hippocampal regions (60μg total protein) were isolated using streptavidin magnetic beads. 100% of the elute of the biotinylated surface fractions (S) were resolved alongside 25% of their corresponding internal fractions (I) and probed for the intracellular protein GAPDH as control for selective isolation of plasma membrane proteins. As expected, GAPDH levels were almost absent in all major hippocampal regions from the biotinylated fraction (S) but present in abundance in the nonbiotinylated fractions (I).

**Figure S4. Examples depicting qRT-PCR amplification plots for Ca_v_1.2 and Ca_v_1.3 gene expression analyses.** No significant age-related changes in mRNA levels were observed for both Ca_v_1.2 (A) and Ca_v_1.3 (B) genes in any of the three major hippocampal regions. Gapdh (C) was used as internal housekeeping control.

**Figure S5. Regional expression of Ca_v_1.2 and Ca_v_1.3 L-type calcium channel proteins in dorsal hippocampus.** Homogenates from each major hippocampal region from young and aged rats were analyzed using semi-quantitative Western blotting techniques and immunoblotted using antibodies against Ca_v_1.2 (CNC1) and Ca_v_1.3 (Hall et al., 2013)(ab144) L-type calcium channel proteins. (A) Representative Western blots comparing regional expression of Ca_v_1.2 and Ca_v_1.3 proteins in CA3, DG, and CA1 of young and aged rats (n=2). (B, C) Quantitation of total L-type calcium channel expression in young and aged rats normalized to GAPDH and relative to CA1 in each condition group. (D) Integrated optical densities for GAPDH in young (n=9; open circles) and aged (n=9; closed circles). Ca_v_1.2 (B) and Ca_v_1.3 (C) channel protein expression was found to be significantly elevated in DG region in both young and aged animals when compared to CA1 and CA3 region. Regional differences in protein levels due to sample loading was discarded as no differences in integrated optical densities were found with our housekeeping protein (GAPDH) when compared between and within regions (A, D). All results were confirmed by repeating the experiments and analysis twice. Unpaired t-test: *p<0.05, **p< 0.005, ***p<0.0001. Data reported as the mean ± SEM.
